# Aetiological Significance of Infectious Stimuli in Kawasaki Disease

**DOI:** 10.3389/fped.2019.00244

**Published:** 2019-06-28

**Authors:** Akihiro Nakamura, Kazuyuki Ikeda, Kenji Hamaoka

**Affiliations:** ^1^Central Research Laboratory, Graduate School of Medical Science, Kyoto Prefectural University of Medicine, Kyoto, Japan; ^2^Department of Pediatrics, Graduate School of Medical Science, Kyoto Prefectural University of Medicine, Kyoto, Japan; ^3^Pediatric Cardiology and Kawasaki Disease Center, Uji-Tokushukai Medical Center, Kyoto, Japan; ^4^Faculty of Life and Medical Sciences, Doshisha University, Kyoto, Japan

**Keywords:** Kawasaki disease, vasculitis, animal models, infection, superantigens, pattern recognition receptors

## Abstract

Kawasaki disease (KD) is a pediatric vasculitis syndrome that is often involves coronary artery lesions (e. g., coronary artery aneurysms). Although its causal factors and entire pathogenesis remain elusive, the available evidence indicates that the pathogenesis of KD is closely associated with dysregulation of immune responses to various viruses or microbes. In this short review, we address several essential aspects of the etiology of KD with respect to the immune response to infectious stimuli: 1) the role of viral infections, 2) the role of bacterial infections and the superantigen hypothesis, 3) involvement of innate immune response including pathogens/microbe-associated molecular patterns and complement pathways, and 4) the influence of genetic background on the response to infectious stimuli. Based on the clinical and experimental evidence, we discuss the possibility that a wide range of microbes and viruses could cause KD through common and distinct immune processes.

## Introduction

Kawasaki disease (KD), named after its discoverer Dr. Tomisaku Kawasaki, is a pediatric vasculitis syndrome which is characterized by clinical manifestations including fever persisting for 5 days or more, swelling of the cervical lymph nodes, conjunctival infection, changes in oral mucosa and the tongue, skin rash, and redness of the palms and soles of the feet (1, 2). Although KD shows a systemic vascular inflammation, the coronary arteries are one of the worst affected sites. Without adequate treatment in the acute phase, approximately 30% of patients exhibit coronary artery lesions (CALs) including coronary arterial dilation, stenosis, and aneurysms (3).

Treatment of KD typically features intravenous immunoglobulin (IVIG) therapy (4). In fact, IVIG has markedly decreased the mortality rate in patients with acute KD. However, a persisting concern is that the disease may impair cardiovascular health in adults with a history of KD. Furthermore, approximately 20% of acute KD patients show a low response to IVIG (5). The therapeutic resistance also results in an increased risk of CALs and future cardiovascular events.

**Figure 1:**
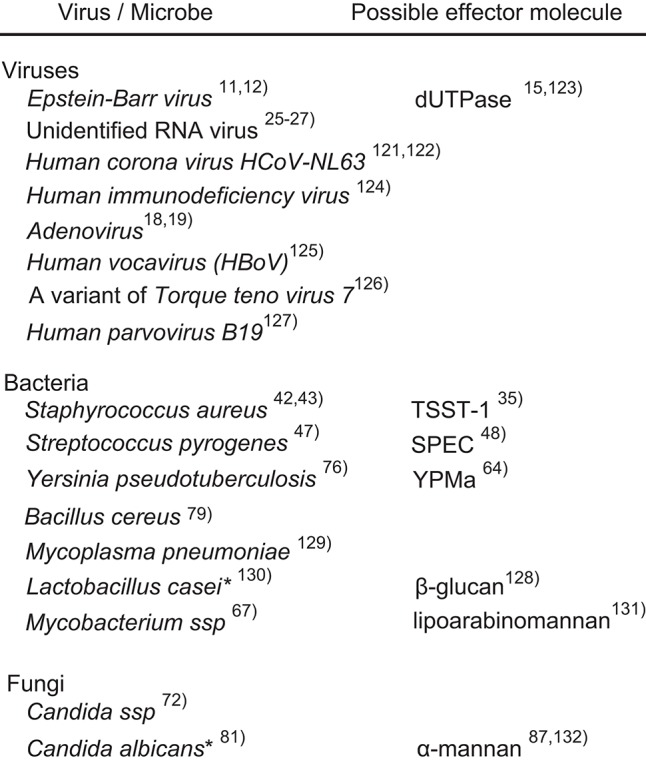
Possible causal microorganisms of Kawasaki disease. Most of the listed microorganisms were identified on the basis of PCR or serological examinations of clinical specimens. The asterisks indicate experimental evidence from animal models, not clinical specimens.

The aetiological mechanism of KD remains unclear and the causal factors are also unknown (6). Although there is no definitive evidence that KD is an infectious disease, recent studies support the view that a dysregulated immune response to a variety of infectious stimuli is likely to contribute to KD pathogenesis (6, 7). Based on these studies, this short review explores the possible relationship between KD and the immune response to various infectious agents (Figure 1).

## Involvement of Viruses in KD Pathogenesis

The incidence of KD exhibits seasonality and outbreaks occurred in Japan in 1979, 1982, and 1986 (8, 9). This has led to the speculation that viral infection may underlie KD pathogenesis. Based on serological and polymerase chain reaction (PCR) based-analyses of clinical specimens, at least 14 species of the virus have been reported to be relevant to KD (10). We consider here three possible candidates: Epstein-Barr virus (EBV), human adenovirus, and a putative KD-associated RNA virus.

### Epstein-Barr Virus

EBV is a type of human herpes virus. Kikuta et al. (11, 12) reported that the EBV DNA sequence was detected in 83% of KD patients and in 18% of control subjects. Chronic active EBV infection sometimes involves CALs, including coronary artery aneurysms (13, 14). Although the pathogenesis of EBV-infection-associated CALs is unclear, it has been demonstrated *in vitro* that deoxyuridine 5'-triphosphate nucleotidohydrolase (dUTPase), an EBV-encoded protein, stimulates monocyte-derived macrophages through Toll-like receptor 2 (TLR2)-dependent signaling (15). This up-regulates the production of interleukin-6 (IL-6) (15), which activates endothelial cells (ECs) and platelets. Contrary evidence has also been presented on the involvement of EBV in KD (16, 17).

### Adenovirus

It has been known that adenoviral infection exhibits seasonal pattern and some symptoms similar to that of KD (18, 19). However, semi-quantitative PCR-based investigations found no association between adenovirus and KD (20). Gene microarray of the blood samples also showed the distinct pattern between KD and adenovirus-infected patients (21). Further study is needed to clarify the involvement of adenoviral infection in KD (22).

### Putative RNA-Associated Virus

Immunoglobulin A (IgA)-producing plasma cells are observed in the affected arterial tissue of KD patients (23, 24). Rowley et al. (25, 26) detected RNA virus-like inclusion bodies in the cytoplasm of bronchoepithelial cells of KD patients. It was detected using synthetic antibodies generated from the alpha and kappa chain-encoding DNA sequences, which were cloned from the affected arterial tissue of KD patients (27). However, the putative KD-associated RNA virus has not been identified yet.

Previous studies suggest that the involvement of viruses in KD is still very controversial. However, a recent transcriptomic study reported the significant up-regulation of a set of type I interferon (INF)-induced genes closely related to cellular antiviral processes in the coronary arteries of KD patients (28). Furthermore, the increased plasma level of C-X-C motif chemokine ligand 10 (CXCL10/IP-10), a representative INF-alpha2a/gamma-inducible protein was recently reported as a promising biomarker for the early acute phase of KD (29). These two studies raise the possibility that KD pathogenesis might be associated with a common immune response to viral infections.

## Bacterial Infection and Superantigens Hypothesis

Superantigens (SAgs) are a group of proteins, which can activate approximately 20% of the T cells in the peripheral blood. SAgs stimulate these cells by forming a bridge between the T-cell receptor and the major histocompatibility complex class II (MHC-II) of antigen presenting cells in the absence of any antigenic peptide presentation (30, 31). This results in the overproduction of pro-inflammatory cytokines, including tumor necrosis factor-alpha (TNF-α), by the activated T cells (32). Human MHC-class II and co-stimulators are also expressed in the endothelial cells (33). In fact, SAgs can directly injure these cells in conjunction with T cells (34, 35) *in vitro*.

SAgs are produced by a wide range of organisms, including bacteria, viruses, fungi, and plants (36–38). The pathological role of SAgs has been well-studied with respect to toxic shock syndrome and scarlet fever, which are caused by *Staphylococcus aureus* and *Streptococcus pyogenes*, respectively (39, 40). KD displays some clinical similarities to these two diseases (41).

### *Staphylococcus aureus* and *Streptococcus pyogenes*

*Staphylococcus aureus* produces a SAg designated TTS toxin-1 (TSST-1), which induces expansion of Vβ2 T cell receptor (TCR)-positive T cells. Some early studies reported the frequent detection of TSST-1-producing *S. aureus* or the specific antibody to the SAg in KD-patients (42, 43). As ECs express MHC-II and co-stimulators, TSST-1 can activate human umbilical vein ECs *in vitro* in the presence of T cells (34). Considering that specific types of vessels (e.g., coronary arteries), are preferentially affected in KD., it might be worth investigating the pathophysiological significance of the role of ECs as a semi-professional antigen-presenting cells in KD (44, 45).

Streptococcal pyrogenic exotoxin C (SPEC) selectively activates Vβ2-bearing T-cell subsets and Vβ6.5-bearing ones (46). The number of Vβ2-/Vβ6.5-bearing T-cell subsets and anti-SPEC antibody levels are increased in the peripheral blood of acute KD patients (47). DNA fragments encoding these SAgs were also significantly more prevalent in the stool of KD patients than in that of non-KD febrile subjects (48), indicating that these bacteria-derived SAgs are involved in KD pathogenesis. *Streptococcus pyogenes* infection occasionally triggers autoimmune heart diseases, such as rheumatic fever. The serum level of IgM-type autoantibody to the cardiac myosin heavy chain, which is highly homologous to group A streptococcal M5 protein (49), was increased in KD patients (50). A variety of autoantibodies has also been detected in KD patients (51–53). Besides molecular mimicry, SAgs could be implicated in the activation of potentially autoreactive peripheral T and B cells (54).

Although the physiological role of circulating follicular T helper cells (cTfh) remains elusive, the cells could be stimulated by SAgs and/or other pathogen-derived molecules. Xu et al. (55) reported that IL-4 and the cTfh2 subpopulation of total cTfh cells significantly increased during the acute phase of KD. Increases in cTfh2 and IL-4 are observed in IgA-vasculitis and IgG4-related disease (IgG4-RD) (56–58). Although the general clinical features of IgG4-RD are apparently dissimilar to those of KD, IgG4-RD occasionally involves abnormalities of large- and intermediate-sized vessels, including coronary arteritis (59).

Despite the foregoing evidence, the involvement of SAgs in KD has not been definitively confirmed. Some studies have independently found no significant elevation of antibodies against *S. aureus* or *S. pyogenes*-derived SAgs in KD patients (60, 61). Although these bacterial-derived SAgs may provide a rationale to understand the pathogenesis of KD, its aetiological significance is still very controversial.

### Yersinia pseudotuberculosis

Based on the similarity in clinical manifestations and the data from some serological studies, it has been argued that *Y. pseudotuberculosis* (YP) infections might be involved in the pathogenesis of KD (62). Some species of YP produce YP-derived mitogens (YPM), which are SAg-like virulence factors (63). Consistent with the increased prevalence of KD in Far East countries, YPM-positive pathogenic YP are also predominantly distributed in these countries and are less frequent in western countries (64). However, in a recent clinical study involving 108 Japanese KD patients, it was found that 90% of patients were negative for antibodies to YP and YPM (65). Vβ2-bearing T-cell subsets be preferentially activated in KD, whereas Vβ3, Vβ9, Vβ13.1, or Vβ13.2-bearing T-cell subsets are activated in YP infection (66).

### Mycobacterium

Reactivation of *Bacillus Calmette-Guérin* (BCG) scar is a well-established clinical manifestation in acute KD, indicating that Mycobacteria or immunologically related pathogens might be involved in KD. Although Mycobacteria have not been isolated from KD patients as a causal pathogen, antibody and CD4^+^/CD8^+^ T-cell clones specific to Mycobacterium heat shock protein 65 have been detected in KD patients (67, 68).

Besides *M. tuberculosis, M. leprae*, and *M. lepromatosis*, non-tuberculous mycobacteria (NTM) can cause self-limited infections in humans. *M. avium complex* (MAC) is a representative NTM. The immune response to MAC infections involves peculiar macrophages, which are characterized by the co-expression of anti-inflammatory M2 macrophage markers (e.g., CD163, IL10) and markers of pro-inflammatory M1 macrophages (e.g., CCR7, IL1β) (69). These cells promote Th17 cell-differentiation (69). Intriguingly, the coronary arteries affected in acute KD also often feature marked infiltration of macrophages, which are negative for CD80 (an M1 marker), and positive for CD163 (an M2 marker) (70). Plasma level of Th17-related cytokine sets are also increased (71). Considering these recent findings and BCG scar reactivation in KD, re-visiting the possible implication of mycobacteria in KD could be warranted.

### Fungi

Although there is no definitive clinical evidence suggesting that fungi are a causal factor for KD, it has been well-established that *Candida albicans* extracts develop KD-like experimental model of vasculitis in mice. Based on the meteorological and enviromentological study, Rodo et al. (72) recently reported that KD is associated with tropospheric winds containing Candida species as a predominant fungus. Similar investigation in Canada also suggested the implication of westerly wind-associated fungi in KD (73).

It might be possible to validate phlogogenic activity of the wind-blown microbes / molecules with established animal models for KD.

### Possible Triggers and Diagnostic Criteria of Kawasaki Disease

Except *Y. pseudotuberculosis* infection (65, 74, 75), few cases satisfy the six diagnostic criteria for Kawasaki disease (KD) among the infectious diseases caused by the aforementioned agents. Considering that genetic background is closely associated with the susceptibility to KD (see section Influence of Genetic Background Affecting Response to Infectious Stimuli), polymorphisms in some specific genes of infected children might affect the clinical futures of the infectious diseases (76). Moreover, it is not exclusive that unidentified agents could be involved in the onset of KD. Caution should be exercised when considering the possible causal agents on the basis of the symptom similarity.

## Aetiological Significance of Innate Immune Response in KD

While innate immunity is the first line of self-defense against infectious agents, it is also accompanied by inflammatory reaction (77). Thus, its inadequate regulation gives rise to inflammatory tissue damage. Accumulating evidence indicates that KD could be associated with dysregulated innate immune response.

### Insight From Experimental Studies With Animal Model for KD

Apart from SAgs, a variety of microbes or virus-derived biomolecules (e.g., lipopolysaccharides, glucans, and nucleotides) can stimulate immune cells or other cell types, including ECs. These biomolecules are designated pathogen-associated molecular patterns (PAMPs) and microbe-associated molecular patterns (MAMPs). PAMPs and MAMPs are recognized by a type of cellular or soluble receptors, which are designated pattern recognition receptors (PRRs) (e.g., TLRs) to trigger innate immune responses, including the production of inflammatory cytokines through intracellular signaling pathways. A possible implication of these PAMPs or MAMPs in KD has been demonstrated in mouse models and more recently in clinical specimens from KD patients (78, 79).

KD-like vasculitis can be induced in rabbit, swine, and mouse models by various methods (80–83). The known genetic background and ease of genetic manipulation have made mouse models the preferred model to investigate molecular pathogenesis of KD and to discover its therapeutic targets (84–88).

In a murine model of vasculitis, *Candida albicans* water-soluble fraction (CAWS) is used as the inducer. Marked inflammatory change is observed in the aortic root, including the coronary arteries in CAWS-treated mice (89, 90). While CCR2 and GM-CSF play an indispensable role in its pathogenesis (89, 90), T and B cells are also involved in the vasculitis (89). Sensitivity and severity of CAWS-induced vasculitis apparently depend on mouse strain, suggesting that genetic background affects its pathogenesis (also see section Influence of Genetic Background Affecting Response to Infectious Stimuli).

Another KD-like murine coronary arteritis model involves induction by *Lactobacillus casei* cell wall extract (LCWE). TLR2-dependent signaling, ILβ-dependent signaling, and CD8^+^ T cells (cytotoxic T cells) play a crucial role in its pathogenesis (91–93).

KD-like coronary arteritis can also be induced in mice by NOD1 ligand, FK565 (94, 95), a synthetic derivative of acylpeptide produced by *Streptomyces olivaceogriseus* (96). The experimental model of vasculitis reportedly underlies the interaction between the CCR2 expressed in monocytes and the CCL2 induced by the NOD1-dependent signaling in EC (78).

It is important to consider whether these models accurately represent the actual molecular mechanisms of KD (97). However, some evidences indicate that the experimental murine vasculitis is relevant to KD pathogenesis. The antibodies to β-glucan, another major CAWS component, was increased in KD patients (98). Regarding findings in CAWS-induced or FK565-induced mouse model, it was also reported that genetic polymorphisms in the CCR3-CCR2-CCR5 gene cluster are associated with KD susceptibility (99). Furthermore, it has been found that lipophilic substances almost identical to MAMPs derived from *Bacillus cereus, Bacillus subtilis, Y. pseudotuberculosis*, and *S. aureus*, have been detected in the serum of KD patients (79). This finding supports the possibility that KD underlies the molecular pathogenesis similar to the aforementioned mouse models. The possible involvement of *Y. pseudotuberculosis* and *S. aureus* in KD has been argued based on other clinical evidence (also see sections *Staphylococcus aureus* and *Streptococcus pyogenes* and *Yersinia pseudotuberculosis*). The collective findings indicate the possible relationship between PAMPs/MAMPs and the aetiological mechanism of KD vasculitis.

### Other Insight to PAMPs/MAMPs Hypothesis in KD

Regarding the implication of PRRs, Huang et al. (100) reported that the CpG sites of TLR genes were reversibly hypomethylated in the peripheral whole blood cells of acute KD patients, resulting in upregulated expression of TLRs. This transient epigenetic change supposedly potentiates the TLR-dependent innate immune response. DNA demethylation in immune cells is also observed in patients infected with mycobacteria and certain viruses (101, 102), possibly implicating these intracellular-living pathogens in KD.

While killer immunoglobulin-like receptor (KIR) expressed in natural killer cells interacts with HLA-I to suppress activation of NK cells, KIR3DL1/2, a subtype of KIR, also recognizes and KIR3DL2 engulfs pathogen-derived CpG-oligodeoxynucleotide (CpG-ODN) to activate TLR9-dependent signaling (103). A recent case-control study of HLA-A and -B genotypes in Caucasian KD patients proposed the hypothesis that high abundance of HLA ligands for KIR3DL1/2 in KD patients interferes with KIR-dependent cellular CpG-ODN sensing to impair effective clearance of pathogens during infection (104). Consequently this impaired clearance might allow expansion of PAMPs/MAMPs. Although accumulating evidence suggests that PAMPs/MAMPs are involved in KD pathogenesis, their pathophysiological significance may be more complex than is currently appreciated.

### Possible Implication of Complement Pathways

Three complement pathways (classical, lectin, and alternative pathway) are major components of the innate immune system against infectious agents. However, their excess activation causes inflammatory tissue injury. Indeed, accumulating evidence suggests that their dysregulated activation underlies the pathogenesis of vascular inflammation and aortic aneurysms (105, 106). Nevertheless, a limited number of studies had been undertaken regarding the involvement of complement pathways in KD (107, 108).

Recently Okuzaki et al. (109) found that ficolin-1, a circulating soluble PRR that is responsible for activating the lectin pathway, was increased in acute KD. They also demonstrated that KD-like murine vasculitis is ameliorated by infusion of an inhibitory antibody to ficolin-1 (110), suggesting that the lectin pathway could participate in KD pathogenesis. The lectin pathway is triggered by activation of mannose-binding lectin-associated serine proteinases (MASPs). In addition to their role in the complement system, MASPs are involved in coagulation and EC activation (111), both of which are closely connected to KD.

Complement systems are rigorously regulated by more than ten protein species to prevent their undesirable excess activation. Genetic polymorphisms of these proteins might affect susceptibility of KD under infectious condition (106, 112).

## Influence of Genetic Background Affecting Response to Infectious Stimuli

While a variety of microorganisms could be causative agents of KD, the prevalence of KD in children is potentially limited, suggesting that the genetic background of an individual likely affects the disease susceptibility. A series of clinical genetic investigations have revealed KD-related gene polymorphisms in more than 20 genes (113), although its aetiological significance is elucidated only in a limited number of these polymorphisms.

Statistical genetic studies identified an SNP associated with KD susceptibility and resistance to IVIG therapy in the gene encoding inositol-triphosphate 3-kinase C (ITPKC), which suppresses T-cell activation and regulates inflammasome activity in macrophages (114, 115). This SNP destabilizes ITPKC mRNA and reduces the cellular level of ITPKC protein (115). KD-associated gene polymorphisms have been discovered in the CASP3 gene, which might also influence the down-regulation of activated immune cells (116). In addition, Onouchi et al. (114) reported KD-associated polymorphisms in the gene encoding ORAI1, a calcium release-activated calcium channel protein 1. Notably, these proteins are also involved in the calcium-dependent nuclear factor-activated T cell (NFAT) pathway (117, 118).which regulates the function of T and B cells. EBV-encoded latent membrane protein 1 (LMP-1) up-regulates ORAI1 (119). Considering that EBV is a possible trigger for KD (see section Epstein-Barr Virus), it is intriguing whether the identified genetic polymorphisms could affect immune response to EBV infection.

The collective molecular genetic data indicate that most genes with KD-associated polymorphisms are responsible for the modulation of inflammatory responses, including T-cell activation, which is compatible with the postulated roles of SAg and PAMPs/MAMPs. This strengthens the possibility that the dysregulated immune response to infectious stimuli underlies the pathogenesis of KD.

Induced pluripotent stem cell technique has been successfully used to establish some human EC lines harboring genetic backgrounds of KD patients (120). Studies with these cells enable the verification of the aetiological significance of individual genetic backgrounds in KD *in vitro*.

## Conclusion

**Figure 2:**
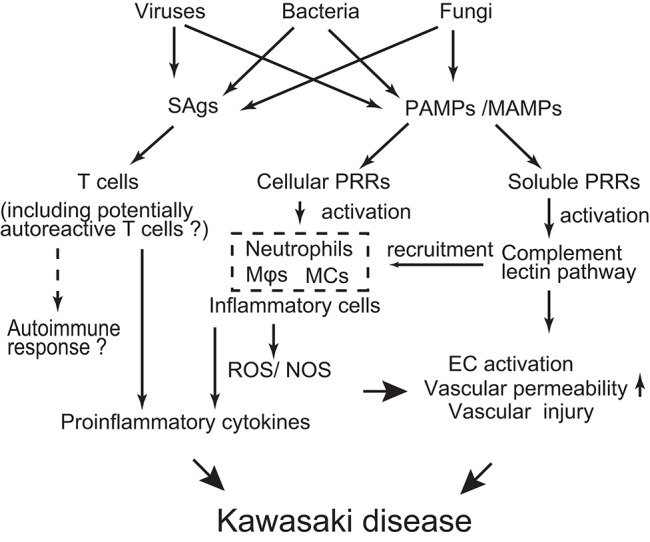
Schematic depicting possible mechanisms of KD pathogenesis. Various infectious agents produce superantigens (SAgs) and pathogen/microbe-associated molecular patterns (PAMPs/MAMPs). SAgs non-specifically activate T cells, probably including potentially autoreactive T cells. PAMPs/MAMPs also stimulate immune cells [e.g., macrophages (Mϕ), dendritic cells (DCs), monocytes (MCs)] and endothelial cells (ECs) through cellular pattern recognition receptors (PRRs) (e.g., TLRs, NOD1, Dectin-1/-2). This stimulation up-regulates production of pro-inflammatory cytokines/chemokines and reactive oxygen/nitrogen species (ROS/NOS), leading to a systemic inflammatory reaction. On the other hand, PAMPs/MAMPs also activate the complement lectin pathway through soluble PRRs (e.g., ficolin-1, mannose binding lectin-2). Activated complement pathways can elicit inflammatory vascular damage through recruitment of innate inflammatory cells and direct injury to ECs. The extent of the inflammatory reaction is influenced by the genetic backgrounds of the individuals, resulting in a limited number of children developing KD in response to infectious stimuli.

Although the etiology of KD is far from being resolved, the evidence collected so far drives the following hypothesis (Figure 2): Diverse pathogens could be potential causative agents of KD. However, such different infectious stimuli converge on a similar/common immune process associated with the activation of T cells, innate immune cells, and ECs. The genetic background of infected children affects the magnitude of the immune responses to develop KD in a limited number of children.

## Author Contributions

The manuscript was written by AN, and it was edited by KI and KH.

### Conflict of Interest Statement

The authors declare that the research was conducted in the absence of any commercial or financial relationships that could be construed as a potential conflict of interest.
